# Supplementary material for: “You’re kind of at war with yourself as a nurse”: Perspectives of inpatient nurses on treating people who present with a comorbid opioid use disorder
Source: PLoS One. 2019 Oct 24;14(10):e0224335. doi: 10.1371/journal.pone.0224335 (PMC6812769; doi:10.1371/journal.pone.0224335)
Supplement: S1 Appendix — (DOCX) [file pone.0224335.s001.docx]

Appendix 1: Nurses Interview Guide

# Introduction

Hi, [Nurse’s Name]. Thank you for speaking with me today. My name is and I am a researcher at Tufts School of Medicine. We are interviewing nurses on inpatient units to assess the attitudes, perceptions, and challenges nurses may encounter when treating patients who have opioid addictions. We are interested in hearing your thoughts on how TMC can support you and other nurses in caring for this patient population. The goal of our research project is to summarize the experiences of nurses in treating patients addicted to opioids and to compile a series of recommendations on ways to address professional concerns nurses may encounter in the workplace.

Firstly, I will be conducting an open-ended interview that will last for no more than 25 minutes. While we will be recording this interview, the audio file will be transcribed and then deleted. The transcript will not contain identifying information nor will it be shared with any individual outside our study team. While the interview will focus on your professional experiences and opinions, the topic of drug abuse and addiction is sensitive. You are free to not answer any question that I may ask and may end the interview at any point.

Then, we hope you could complete a brief paper questionnaire which will take less than 5 minutes. These responses will also remain anonymous. You are free to skip over any question or choose not to complete the survey. We will create a study ID number that links the demographic form to the survey and interview transcript, although there is no information collected that will link back to you personally. All information you provide will be stored on a secure drive accessible only to study team members for the purpose of data analysis.

Participation is completely voluntary and you will not be penalized in any way for refusing to participate. If you have additional questions or concerns, feel free to reach out to our Principal Investigator, Dr. Alysse Wurcel at awurcel@tuftsmedicalcenter.org. You may also reach out to the IRB Administrator for this project, Dr. Lara Sloboda at the Office of the Institutional Review Board at (617) 627-3417.

Do you agree to participate? If YES, continue...

1. Can you discuss your experiences treating patients who are dependent on opioids?
2. What are the greatest challenges you face in caring for patients who are or who you suspect are dependent on opioids? If you practiced in other hospitals or geographic areas, do you think these challenges differ?
3. What do you think are the greatest challenges patients who are dependent on opioids face in the hospital setting?
4. What kind of training have you received on how to treat patients who are dependent on opioids? Who conducted or sponsored this training and what did it emphasize?
5. How sufficient was this training?
6. In what ways do you think the hospital could enhance the care of patients who are dependent on opioids?
7. In what ways could communication be improved between providers (e.g. doctors, nurses, pharmacists, etc.) regarding treatment of patients with comorbid opioid dependence?
8. Can you tell us how “staff splitting” affects your ability to work with patients who misuse opioids and ways that this issue could be mitigated to help with professional relationships in the hospital?
9. What established policies are you aware of specific to handling situations in which a patient who is dependent on opioids is displaying aggressive or inappropriate behavior?
10. How might your personal experiences influence your treatment or perception of patients who are dependent on opioids?
11. How do your experiences working with people who misuse opioids influence your personal experiences with others in your life?
12. Would you be interested in learning more about addiction and patients with opioid dependence through CEU credits? If so, what type of CEU eligible seminars or events would you like for TMC to offer (i.e. specific topics, speakers, trainings, etc.)?
13. Do you have any final thoughts or comments you would like to share?
